# The usefulness of a three-dimensional printed segmental scapula prosthesis for recovering shoulder function in a patient with scapula chondrosarcoma: A case report

**DOI:** 10.1097/MD.0000000000024817

**Published:** 2021-02-26

**Authors:** Jong Hoon Park, Hae Woon Jung, Woo Young Jang

**Affiliations:** a Department of Orthopedic Surgery, Anam Hospital, Korea University College of Medicine; b Department of Pediatrics, Kyung Hee University Medical Center, Seoul, Republic of Korea.

**Keywords:** chondrosarcoma, shoulder function, three-dimensional printing implant

## Abstract

**Rationale:**

Localized chondrosarcoma of the scapula has a favorable long-term survival outcome. Therefore, recovery of shoulder function after surgery is important in middle-aged patients. Currently, three-dimensional (3-D) printing implants can be applied for personalized limb salvage surgery.

**Patient concerns:**

A 41-year-old woman with a palpable scapular area presented with shoulder pain for 3 months, which was aggravated during shoulder exercise.

**Diagnoses:**

**C**hondrosarcoma at left scapular (Malawer S1, Enniking II B, and grade II chondrosarcoma).

**Interventions:**

Wide excision for a localized chondrosarcoma at the infrascapular lesion was performed and the resected muscles around the scapula were repaired with a 3-D printed segmental scapula prosthesis for recovery of shoulder function.

**Outcomes:**

The affected shoulder achieved satisfactory function after operation using the 3-D printed segmental scapula prosthesis at 1 year 6 months after the operation.

**Lessons:**

The 3-D printed segmental scapula prosthesis is a useful method for shoulder functional recovery in patients with scapula chondrosarcoma.

## 1 Introduction

Chondrosarcoma is the second most common primary bone malignancy.^[1]^ Chondrosarcoma of the flat bones differs from chondrosarcoma arising within the long bones. Surgical resection is the key treatment for chondrosarcoma because it is resistant to chemotherapy and radiotherapy.^[2]^ According to a previous report,^[3]^ simple scapulectomy, either total or subtotal, can achieve relatively good functional outcomes. However, the reported shoulder function in the literature was commonly evaluated using the questionnaire for shoulder function and range of motion (ROM). No reports have been made regarding the measurement of muscle strength and endurance around shoulder muscles in patients with scapular chondrosarcoma. Herein, we present the case of a patient with localized chondrosarcoma at an infrascapular lesion who underwent reconstruction using a three-dimensional (3-D) printed segmental scapula prosthesis after subtotal scapulectomy. At 1 year 6 months after the operation, she was evaluated for shoulder function using isokinetic muscle testing. The patient was informed that data concerning the case would be submitted for publication of the case, and she provided consent.

## 2 Case presentation

**Figure 1:**
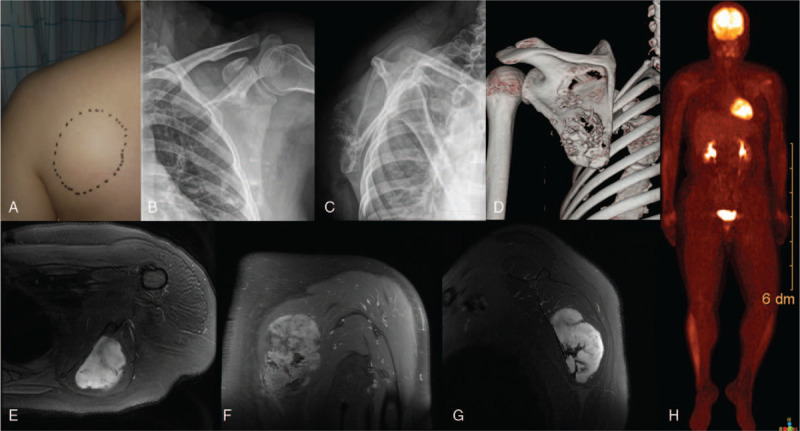
A 41-year-old woman presented with a palpable mass in the scapular area (A) and a bone lesion at the scapula on plain radiography (B and C). A lobulating contoured heterogeneous enhancing mass with internal calcifications was identified in the left infrascapularis muscle with adjacent scapula destruction (D–G). No evidence of distant metastasis can be found (H).

A 41-year-old woman with a palpable scapular area presented with shoulder pain for 3 months, which was aggravated during shoulder exercise (Fig. 1A). Physical examination revealed no limited ROM of the shoulder, but pain occurred when external rotation (ER) of the shoulder was performed at 90° abduction position. Plain radiographs showed an irregular shadow with a bone lesion on the scapula, located in the S1 region according to Malawer's classification^[4]^ (Fig. 1B and C). Computed tomography (CT) and magnetic resonance imaging revealed an approximately 8.9- × 7.9- × 4.2-cm-sized lobulating contoured heterogeneous enhancing mass with internal calcifications in the left infrascapularis muscle with an adjacent scapula destruction (Fig. 1D–G). No abnormalities were found adjacent to the ribs, humerus, and thoracic vertebra. Positron emission tomography-CT revealed the radioactivity concentration in the left scapular area with abnormal mineral metabolism in the bones and no evidence of distant metastasis (Fig. 1H). Biopsy indicated grade II chondrosarcoma. The diagnosis was as follows: chondrosarcoma in the left scapular, Malawer S1, Enniking II B, and grade II chondrosarcoma.

### 2.1 Interventions

**Figure 2:**
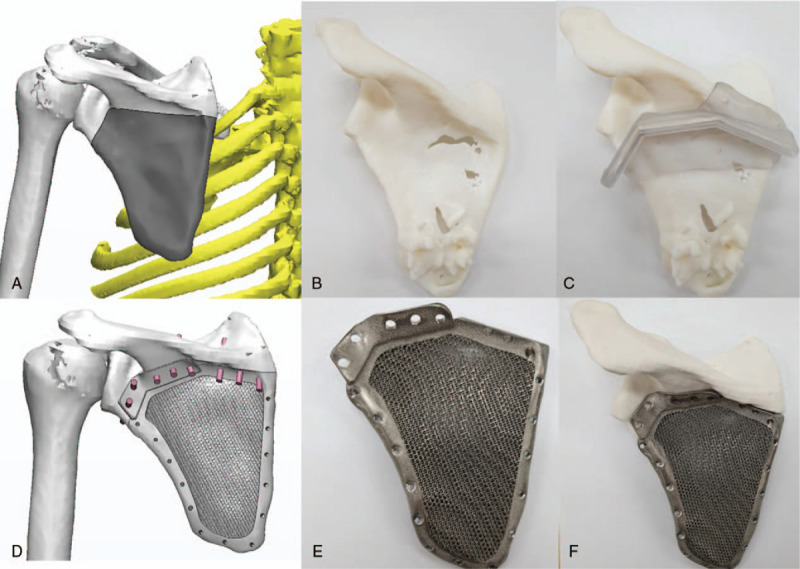
Three-dimensional printing for preoperative planning and surgical simulation is performed.

Considering the favorable survival outcome of the scapular chondrosarcoma and the invasion only in the S1 region, which allowed for an easily negative margin and for the greatest likelihood of restoring limb function in middle-aged patients, partial scapula replacement after tumor resection was agreed upon as the treatment protocol. For segmental scapula prosthesis, CT images of his left shoulder were obtained with 1-mm slices. The Digital Imaging and Communication in Medicine files of the images were then uploaded to a software program (Mimics 19.0, Materialise Company, Belgium) to acquire a virtual 3-D model of his left total scapula by threshold segmentation and region growth processing. A mirror model of the scapula from the healthy side was used to obtain a prosthesis with the same size and shape as the original removed portion. To remove the chondrosarcoma quickly and accurately, an osteotomy guide was designed and printed (Fig. 2A–C).

**Figure 3:**
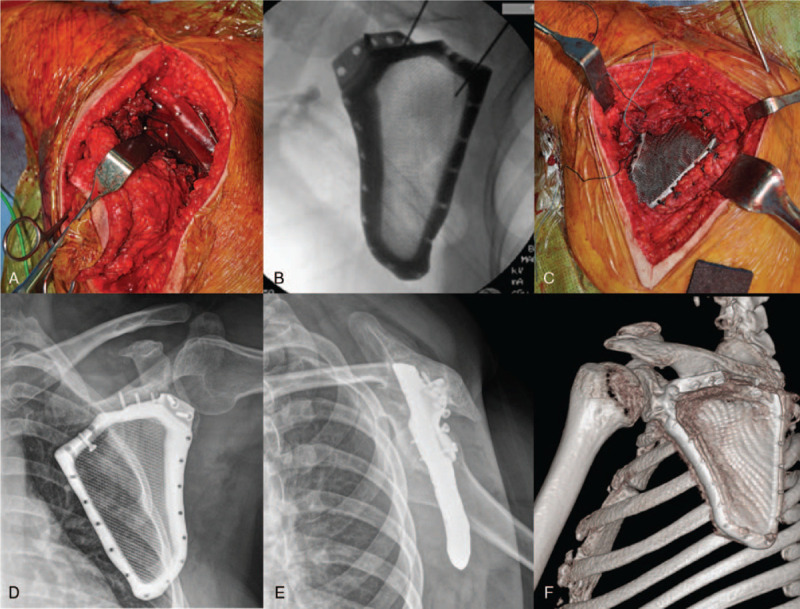
The osteotomy guide to the spine of the scapula and removal of the infrascapular area (A). After implant fixation to the retained scapula by using intraoperative fluoroscopy (B), the surrounding muscle tissue is sutured to the prosthesis through the holes at the edge of the prosthesis (C). The postoperative plain radiographs (D and E) and computed tomography images (F) show good match between the printed segmental scapula prosthesis and the retained scapula.

Intraoperatively, the patient was placed under general anesthesia in the prone position. While attaining adequate exposure of the tumor tissue, we attached the osteotomy guide to the spine of the scapula and removed the infrascapular area (Fig. 3A). The rhomboid, infraspinatus, and teres muscles along the medial walls of the scapula were stripped and resected in an order based on the principles of wide resection. The intraoperative fluoroscopy image indicated a good match between the printed segmental scapula prosthesis and the retained scapula (Fig. 3B). The printed titanium alloy segmental scapula prosthesis was combined with the retained scapula via a 3-D printed contoured plate and 3.5-mm screws. Finally, we sutured the infraspinatus, teres minor muscle, and surrounding muscle tissue to the prosthesis through the holes at the edge of the prosthesis (Fig. 3C).

For 4 weeks after the operation, a shoulder abduction brace was applied in the functional position of the external support. Shoulder functional training was performed for 2 months after removal of the abduction brace. At 3 months after the surgery, the patient returned to her routine life as a mother with a 2-year-old girl and kindergarten teacher.

### 2.2 Outcomes

**Figure 4:**
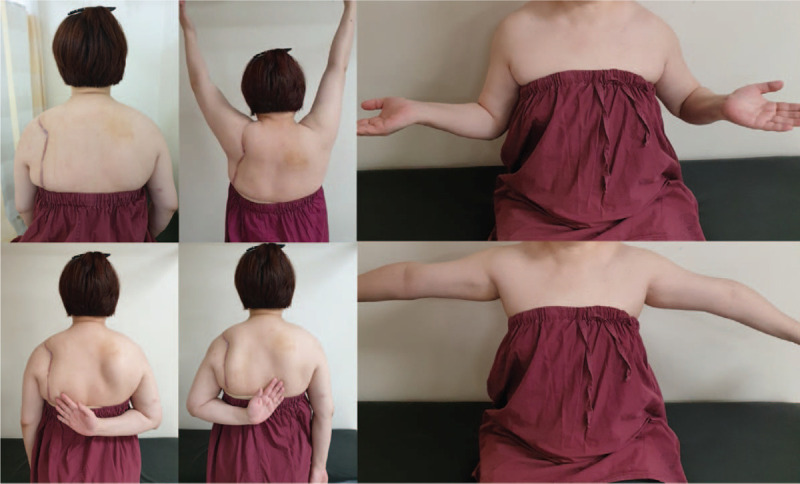
The patient shows good shoulder range of motion at 1 year 6 months after the operation.

At 1 year 6 months after the operation, she showed good shoulder ROM (further flexion: 150°, abduction: 90°, ER: 60, and internal rotation [IR] at back: T7; Fig. 4). No scapula dyskinesia was observed in the affected shoulder. Isokinetic muscle testing was performed to determine the muscle strength and endurance. The IR and ER of the shoulders of the subjects and controls were evaluated with isokinetic muscle testing using Biodex Multi-Joint System 4 (Biodex Medical System Inc., Shirley, NY). The isokinetic tests were performed five times, repetitively, for IR and ER at a speed of 180°/sec (concentric/concentric contraction) for the measurements of muscle function. Furthermore, a handheld dynamometer (CompuFET; Hoggan Health Industries, West Jordan, UT) was used to evaluate shoulder scaption strength.^[5]^

The muscle strength of the affected shoulder was compared with that of the shoulder on the unaffected side as follows: peak torque/body weight: IR, 33.3 Nm kg^−1^ × 100 versus 39.9 Nm kg^−1^ × 100; ER, 13.7 Nm kg^−1^ × 100 versus 20.8 Nm kg^−1^ × 100. The muscle strength ratio between the ER and IR was 41.3 in the affected shoulder and 52.2 in the unaffected shoulder. The muscle strengths of further flexion and abduction on the affected side compared with those on the unaffected side were as follows: 11.6 N versus 17 N and 10.1 N versus 16 N, respectively. The muscle endurance of the affected shoulders compared with that of the shoulders on the unaffected side was as follows: 412.5 J versus 642.5 J for IR and 76.1 J versus 255.9 J for ER. These results indicate that the ER muscle strength and endurance were functionally maintained. The Musculoskeletal Tumor Society score^[6]^ was 21; Constant score, 71; and University of California at Los Angeles shoulder score, 29. We found no evidence of metastasis at the last follow-up.

## 3 Discussion

Chondrosarcoma is a malignant bone tumor that accounts for 20% to 25% of all bone sarcomas.^[1]^ Typically, chondrosarcomas are found in middle-aged patients. The most common locations were the pelvis, proximal femur, distal femur, and scapula. The histological grade of chondrosarcoma correlates with survival. Anatomically, localized chondrosarcoma of the scapula had a favorable long-term survival outcome because of the relatively easy achievement of wide surgical margins with limb-sparing surgery.^[7]^ Therefore, recovery of shoulder function after surgery is important in middle-aged patients. In this study, the 3-D printed segmental scapula prosthesis showed successful outcomes in a middle-aged patient with grade 2 chondrosarcoma.

3-D printed implants have been used mainly in orthopedic oncology.^[8,9]^ Wong et al^[8]^ reported a patient with pelvic chondrosarcoma of the anterior column that was treated with patient-specific instrumentation and a custom-made titanium monoblock pelvic implant. Imanishi and Choong^[9]^ reported the case of a patient with grade 2 chondrosarcoma of the calcaneus who underwent calcaneus replacement surgery with a 3-D printed titanium implant. The use 3-D printed scapular implants have also been reported. Dong et al^[10]^ reported that a patient was treated using a 3-D printed polyetheretherketone total scapula prosthesis for scapular benign fibrous histiocytoma. The patient was satisfied with the results of the surgery and showed a good functional score (Constant score: 68 points). Deng et al^[11]^ reported a patient with grade 1 chondrosarcoma treated with a 3-D printed segmental scapula prosthesis. Although the tumor was located at Malawer S1, it involved both the supraspinatus and infraspinatus lesions, which means that all rotator cuff muscles were resected during the operation. Nevertheless, they described that the patient was in good condition with good functional score (Musculoskeletal Tumor Society score: 28 points). However, no reports have been made regarding the measurement of muscle strength and endurance around the shoulder.

In this study, the patient showed 60% muscle strength of the ER of the affected shoulder when compared with that of the shoulder on the unaffected side and could carry her 3-year-old girl. However, the muscle endurance of the ER of the affected shoulder was 30% as compared with that of the shoulder on the unaffected side. Muscle endurance is defined as continued capability in response to repetitive muscle contraction activity^[12]^; thus, low endurance capability relates to muscle fatigue.^[13]^ On the basis of the results of a previous study,^[14]^ we believe that muscle endurance may increase as muscle strength increases. Furthermore, the affected shoulder abduction strength, which mainly correlated with the supraspinatus and infraspinatus muscles, was maintained at approximately 63% when compared with that of the unaffected side. Maintaining rotator cuff muscles for shoulder function has been reported because these muscles contribute to the formation of the glenohumeral joint, which aids in shoulder joint motion and stability.^[15]^ The infraspinatus and teres minor are involved in anterior-posterior force balance, as the infraspinatus provides the posterior force and the subscapular provides the anterior force, which is called force-couple activation.^[16]^ In this study, considering the life expectancy and activity of the patient, the risk of developing shoulder dysfunction in the future is high if the force-couple activation is deficient. Therefore, we applied the 3-D printed segmental scapula prosthesis to maintain the force-couple activation of the shoulder.

## 4 Conclusion

The 3-D printed segmental scapula prosthesis is a useful method for shoulder functional recovery in patients with scapula chondrosarcoma. Surgeons should keep in mind 3-D printed scapula implants in the planning for the management for scapular chondrosarcoma.

## Acknowledgments

We appreciate cooperation of Kwon Min Hyeok and Lee Jun Hyung at the Cubelabs, Inc. for the 3D printing.

## Author contributions

**Conceptualization:** Hae Woon Jung.

**Formal analysis:** Hae Woon Jung.

**Funding acquisition:** Woo Young Jang.

**Investigation:** Jong Hoon Park.

**Supervision:** Jong Hoon Park, Woo Young Jang.

**Validation:** Jong Hoon Park, Woo Young Jang.

**Visualization:** Hae Woon Jung.

**Writing – original draft:** Jong Hoon Park, Hae Woon Jung, Woo Young Jang.

**Writing – review & editing:** Jong Hoon Park, Woo Young Jang.
